# *In vitro* β-catenin attenuation by a mefloquine-loaded core–shell nano emulsion strategy to suppress liver cancer cells

**DOI:** 10.1039/d4na00547c

**Published:** 2024-11-27

**Authors:** Priyadarshini Mohapatra, Natarajan Chandrasekaran

**Affiliations:** a ICMR-SRF, Centre for Nanobiotechnology, Vellore Institute of Technology Vellore 632014 India; b Centre for Nanobiotechnology, VIT University Vellore 632 014 India nchandrasekaran@vit.ac.in Nchandra40@hotmail.com +91-416-2243092 +91-416-220-2879

## Abstract

Liver cancer, with its robust metastatic propensity, imposes a substantial global health burden of around 800 000 new cases annually. Mutations in the Wnt/β-catenin pathway genes are common in liver cancer, driving over 80% of cases. Targeting this pathway could potentially lead to better treatments. The aim of the present study was to develop a novel strategy for targeting the Wnt/β-catenin pathway while blocking the growth and division, of liver cancer cells and downregulating gene expression. This was achieved by formulating a repurposed drug (mefloquine)-loaded garlic nano-emulsion (GNE) with gold nanoparticles (GNPs) as a core–shell nano-emulsion (MQ/GNE-GNP). The biocompatible core–shell nano-emulsion (MQ/GNE-GNP) exhibited a size distribution in the range of 50–100 nm, high stability, excellent hydrophilicity, good biosafety, and sustained release. Human liver cancer cells were exposed to MQ/GNE, GNPs, and MQ/GNE-GNP at varying concentrations, and the effects were assessed through analysis of the cytotoxicity, reactive oxygen species, cell death, cell cycle analysis, and gene expression studies. It was found that MQ/GNE-GNP arrested HepG2 cells in the sub G0/G1phase and induced apoptosis. The anticancer efficacy of the core–shell nano-emulsion (MQ/GNE-GNP) resulted in higher cell death in the AO/PI staining studies, demonstrating its greater anticancer efficacy. The administration of MQ/GNE-GNP downregulated the overall expression of nuclear β-catenin, thereby suppressing the Wnt/β-catenin pathway. The protein expression level of Wnt 1 was upregulated, while β-catenin expression was significantly decreased. The core–shell nano-emulsion, incorporating a repurposed drug, could disrupt the β-catenin connections in the Wnt/β-catenin pathway. In conclusion, MQ/GNE-GNP could be a promising core–shell nano emulsion for the effective treatment of liver cancer by targeting the Wnt/β-catenin pathway.

## Abstract 2

Liver cancer, with 800 000 new cases annually, is driven by Wnt/β-catenin pathway mutations in over 80% of cases. This study aims to develop a novel strategy to target this pathway, inhibit cancer cell growth and downregulate oncogenic gene expression.

## Graphical abstract

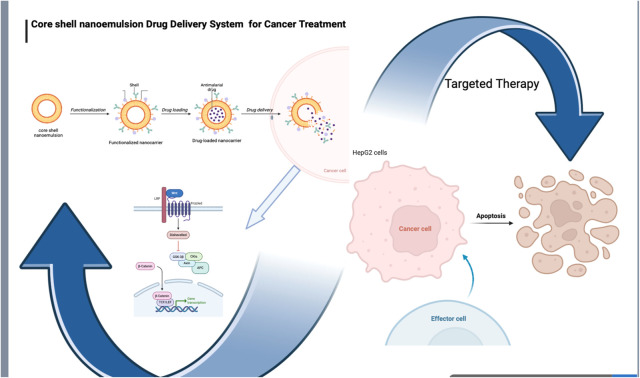

## Introduction

The International Agency for Research on Cancer estimates that in 2020, there were approximately 19.3 million new cancer diagnoses and around 10.0 million cancer-related deaths.^1,2^ Liver cancer, known for its robust metastatic potential, contributes substantially to this disease burden, with approximately 800 000 new cases reported annually across the globe.^3^ Among primary malignancies, hepatocellular carcinoma (HCC) has the greatest incidence and fatality rate. Since liver cirrhosis is a well-known significant cause of HCC, chronic hepatitis B and C viruses are thought to be the key etiologies driving liver cirrhosis.^4^ Even with major advancements in treatment approaches, the 5 year survival rate for individuals with HCC remains low. Hepatocellular carcinoma (HCC) is one of the most common malignancies in humans and is responsible for the second-highest cancer-related deaths worldwide. With a high malignancy, aggressive nature, and dismal prognosis, primary liver cancer ranks among the top three malignant tumours in China. The Wnt/β-catenin pathway drives liver cancer progression, with genomic studies revealing mutations in over 80% of patients.^5^ It has also been found that β-catenin accumulation, a hallmark of this pathway's dysregulation, fuels hepatocellular carcinoma (HCC) and cholangiocarcinoma (CCA). Both tumour types exploit the canonical Wnt signalling, activating β-catenin and promoting oncogenesis.^6^ Consequently, targeting β-catenin has emerged as a promising therapeutic strategy to mitigate liver cancer risk and enhance patient survival. Efforts to develop β-catenin inhibitors possess considerable potential for advancing liver cancer treatment.^7^ The canonical Wnt signalling pathway involves the activation of β-catenin, leading to its translocation into the nucleus and subsequent regulation of target gene expression. Dysregulation of this pathway, particularly aberrant β-catenin activation, is a hallmark of liver cancer pathogenesis.^8^

For liver cancer, there are several treatment options available, including immunotherapy, targeted therapy, radiation, chemotherapy, and surgery.^9^ However, the drug used can have severe adverse effects, including hair loss, vomiting, diarrhoea, and deadly cardiotoxicity, restricting how often treatment can be applied. It has been documented that several targeted moieties, such as vitamins, aptamers, polysaccharides, peptides, and antibodies, accumulate in the liver. As a result, some investigations have employed these ligands to specifically target hepatocarcinoma. Passive and active targeting are the two methods available for achieving nanocarriers targeting.^10^ The field of nanoparticle research has witnessed substantial growth in recent years, with more than half of the 91 000 articles indexed under “nanoparticle” in PubMed as of November 2019 being published since 2010. This trend underscores the exponential rise in research endeavours focused on the investigation and utilization of nanoparticles.^11^ Targeted nanotechnology might offer a special strategy for treatment. For instance, nanocarriers can be effectively localized into the tumour microenvironment through passive targeting.^12^ Using many ligands to boost the targeting system's efficiency and selectivity is a more modern strategy for active targeting.^13^ Compared to conventional drug-delivery techniques, nanocarrier drug-delivery systems offer a longer *in vivo* half-life and less systemic toxicity.^14^ As delivery strategies for the treatment of malignant tumours, nano-emulsions, nanoparticles, and core–shell nano-emulsions as broad nano-carriers are currently widely used.^15^ Recent developments in drug delivery have led to the emergence of core–shell nano-emulsions that have various advantageous properties, including designed drug release and a core–shell structure.^16^ A more stable delivery system was developed in a core–shell nano-emulsion delivery system by co-loading a size-based nanoparticle shell onto a nano-emulsion containing a drug in the core.^17^ Core–shell nano delivery systems have emerged as a research hotspot in the field of pharmaceutics due to their benefits, which include controlled drug release, adjustable surface structure, high efficacy, and outstanding biocompatibility.^18^ Compared to conventional nanoparticle delivery systems, the therapeutic efficacy of the drug is significantly increased by loading one drug into the core and co-loading a particle shell into the core–shell nano-formulation delivery system.^19^ To the best of our knowledge, no research has yet been done on the use of core–shell nano-emulsion delivery systems to deliver malaria drugs that target the Wnt/β-catenin pathway to treat HCC.

Nano-carriers offer a solution for encapsulating poorly soluble drugs and modifying their distribution.^20,21^ MEF, a promising candidate, exhibits low oral bioavailability (39%) due to its high lipophilicity (log *P* = 5.04) and poor water solubility (4 mg ml^−1^).^22^ Efforts are underway to improve MEF's solubility and bioavailability. Poor water solubility remains a significant limitation for many drugs, hampering their absorption.^23,24^ In addition to having many therapeutic benefits, garlic essential oil (GEO)(*Allium sativum* L.) is popularly used in traditional cuisine. The advantages of garlic's several organosulfur compounds (OSCs) have been emphasized by recent studies. In terms of noble metals, gold nanoparticles (GNPs) have drawn a lot of interest due to their exceptional physicochemical and optical characteristics.^25^ The current study's objective was to repurpose an FDA-approved drug through nanotechnology to functionally target the suppression of the oncogenic Wnt signalling/β-catenin. The repurposing drug screening was broadened through nanotechnology and the current research work aimed to formulate and characterize MQ/GNE as a shell and GNP as the core to make a MQ/GNE-GNP core–shell nano-emulsion for targeting the Wnt/β-catenin pathway in liver cancer.

## 2. Materials and method-

### 2.1 Cell lines and chemicals

The HepG2 cell line was purchased from the National Centre for Cell Science in Pune, India. HepG2 cells are a widely used human liver cancer cell line derived from a 15 year-old male with hepatocellular carcinoma. They are commonly used in biomedical research, especially for studying liver function, liver diseases, drug metabolism, toxicity, and cancer biology. HepG2 cells are derived from liver cancer and are frequently used to study hepatocellular carcinoma, the most common type of primary liver cancer. The Hep G2 cell line was maintained using DMEM media supplemented with 8–10% foetal bovine serum (FBS), and 50–100 g ml^−1^ of pen-strep antibiotics at 37 °C and 5% CO_2_ in a humid environment. The cell line was regularly maintained and observed to make sure it was in good enough condition for use in the research. Garlic essential oil (GEO) (Reagent plus 99.9% purity), mefloquine hydrochloride (>98%, HPLC powder, *M*w = 414.77 g mol^−1^), and 2′,7′-dichlorofluorescein diacetate (DCFH-DA) were purchased from Sigma-Aldrich (Bangalore, India). Brij 35 (polyoxymethylene lauryl ether)(*M*w = 1199 g mol^−1^) was procured from SRL, India. Cremophor® EL (polyoxyl 35 castor oil) nitrocellulose membrane, Dulbecco's modified eagle's medium (DMEM), minimum essential medium Eagle (MEM), DMSO (dimethyl sulfoxide), MTT (3-[4,5-dimethylthiazol-2-yl]−2,5-diphenyltetrazolium bromide)), trypan blue, propidium-iodide (a fluorescent dye), DPBS, 2% paraformaldehyde, and 1× PBS were purchased from Hi-Media Pvt. Ltd (Mumbai, India). Fetal bovine serum (FBS), trypsin, penicillin–streptomycin (an antibiotic), phosphate-buffered saline (PBS, pH 7.4), and acridine orange solution were obtained from Gibco (ThermoFisher Scientific, India). The primary antibodies β-catenin and Wnt1 Rabbit Anti-Human IgG were obtained from Abclonal. The secondary antibody, FITC Goat Anti-Rabbit IgG antibody, was bought from BD Biosciences. All the above chemicals were of analytical grade. Ultra-pure water was used as obtained from a Cascada™ bio-water system (Pall Corporation, USA) with a resistivity of 18.2 MΩ cm. The analysis software used included ImageJ (Fiji) software, version 1.53c, and FlowJo X 10.0.7.

### 2.2 Optimization/synthesis of the core–shell nano-emulsion (MQ/GNE-GNP)

The deposition of cationic gold nanoparticles (GNPs) onto the anionically charged drug-loaded nano-emulsion core was the key to the fabrication of the core–shell nanomedicine, which was achieved by a two-step spontaneous emulsification/nanoprecipitation approach.^26^ Briefly, the recipe involved making a strong garlic emulsion that contained mefloquine, which showed good stability for longer than six months. Next, several conditions were used to create gold nanoparticles, including changes in the temperature, time, and concentration, resulting in distinct nanoparticle sizes. A combination of the gold nanoparticle shell and the drug-loaded nano-emulsion core was carefully mixed on a magnetic stirrer running between 150 and 200 rpm.^17^ A promising path for biomedical applications was provided by the complex interaction between the drug-loaded nano-emulsion and size-dependent gold nanoparticles in the ensuing core–shell nanomedicine.^18^ As a result, various ratios of the core–shell solution were added, while keeping the other parameters the same. The condition using a 200 rpm magnetic stirrer for 15 min, room temperature, and a final material ratio of 1 : 2 was selected for the further experiments. A spontaneous technique was used to add the gold nanoparticle solution to the emulsions dropwise while stirring continuously until a clear nanosuspension was formed.^27^ The resultant nanoparticle suspension was then subjected to a series of temperature treatments in order to determine its thermostability, including storage at room temperature, −80 °C, 50 °C, and 4 °C.^28^ The suspension was then allowed to settle at room temperature in order to conduct lengthy stability tests and for thorough characterization.^29^ This carefully thought-out process aimed to clarify the impact of various ratios and temperature ranges on the stability and characteristics of the core–shell nanosuspension, providing insightful information for its possible uses.

### 2.3 Characterization of the core–shell nano-emulsion

#### 2.3.1 Determination of the droplet size and polydispersity index

The core–shell nano-emulsion droplet size was ascertained using a DLS system (SZ-100, Horiba, Japan). To lessen the different scattering effects, all the clear formulations from the overnight process were diluted in a 1 : 4 ratio with water before the final reading, and each set of data was verified in triplicate.^30^ We used laser temperature to evaluate the droplets' electrophilic mobility. Doppler frequency shifts in the scattered laser light were used to measure the droplet size and, subsequently, the polydispersity index.^31^

#### 2.3.2 Determination of the zeta potential

The electrophoretic mobility of the particles in the sample was determined by assessing the *ζ* potential (mV) of the core–shell nano-emulsions. Employing specialized software, the specimens were carefully positioned within a transparent zeta cell and quantified utilizing an SZ-100 Horiba nanoparticle analyzer.^13^

#### 2.3.3 Stability of the formulated core–shell nano-emulsions (CNEs)

The core–shell nano-emulsions' stability was evaluated by centrifugation at 1400*g* rpm for half an hour. The identified optimal surfactant ratio for the non-emulsion formulation was assessed for stability over a duration of one to six months under room temperature conditions.^32^ By focusing on the clarity, surfactant content, and long-term stability, this methodical technique aimed to identify the dynamic behaviours of the core–shell nano-emulsions to improving pharmaceutical formulations.^33^

#### 2.3.4 Physiochemical properties of the core–shell nano-emulsions

A thorough physicochemical property investigation using specialist approaches was applied for analysis of the core–shell nano-emulsions. Using a pH meter (Mark VI, Systronic, Ahmedabad, India), the emulsions' pH was determined. Following the protocol described in ref. 34, turbidity measurements were performed at 600 nm using a UV spectrophotometer (U2910, Hitachi), with MilliQ water acting as the blank reference for baseline calibration. Moreover, water conductivity was used as a benchmark reference while measuring the conductivity of the core–shell nano-emulsions using a conductivity meter (CM180, Elico, Hyderabad, India). These scientific methods can help enable a greater understanding of the complex physical and chemical properties of the core–shell nano-emulsion more deeply.^35^

#### 2.3.5 TEM analysis

Transmission electron microscopy (TEM) imaging at an accelerating voltage of 200 kV (HR-TEM) was carried out using a Tecnai F20 microscope from FEI (JEOL JEM 2100, Japan). One drop of the core–shell nano-emulsion^36^ was placed on to a copper grid, which was then vacuum-dried. This investigation was conducted to determine the ideal core–shell nano-emulsion.

#### 2.3.6 Fourier transmission spectroscopy

Fourier transform infrared spectroscopy (FTIR) was used to examine the produced core–shell nano-formulation, which included a MEF-loaded garlic nano-emulsion coated with GNPs (MEF-GNE/GNP). An FT/IR-6800 FTIR spectrophotometer (Jasco, India) was used for the measurements and analysis, and its spectral range was 400–4000 cm^−1^.^37^ The core–shell nano-formulation's molecular vibrations and interactions may be better understood thanks to this analytical approach, which can also offer comprehensive insights into the structure and chemical makeup of a material.^38^

### 2.4 Drug-release kinetics

Simulated intestinal fluid (SIF) and simulated bodily fluid (SBF) were used to evaluate the core–shell nano-emulsion (MEF-loaded garlic oil nano-emulsion coated with GNP). The USA Pharmacopeia was used to create the two body fluids. The drug^35^ release was aided by Franz diffusion apparatus. A cellulose nitrate dialysis membrane divided the two compartments in the setup, which consisted of a donor compartment and a 5 ml recipient compartment that were both maintained at room temperature.^38^ The release of MEF-GNE/GNP was measured at 284 nm for the first hour at 15 min intervals, and then for the next 12 h (max value). The samples from the recipient compartment were removed, and new fluid was added to maintain a consistent volume of fluid overall.^39^

### 2.5 Cytotoxic effect of the core–shell nano-emulsion

The effects of changing the concentration of different volumes of MEF-GNE/GNP were investigated using the previously outlined approach. An MTT assay kit (3-4,5-dimethylthiazol-2-yl)-2,5-diphenyl tetrazolium bromide) obtained from Hi-media (India) was used to assess the viability of the HepG2 cell lines (L. Chen *et al.*, 2020). A 96-well plate was seeded with 1 × 10^5^ cells per well, and the cells were treated for 24 h with varying doses of core–shell nano-emulsions of MQ/GNE-GNP (25 to 100 μl ml^−1^) for obtaining the IC 50 values and one particular concentration of MQ/GNE, GNPs, and MQ/GNE-GNP for assessing their cytotoxic effects against HepG2 and HACAT cell lines. Here, 20 μl of MTT (5 mg ml^−1^ stock concentration) was added to each well and incubated for 3 h after the predetermined incubation period. After the medium was withdrawn, 200 μl of DMSO was added to each well to dissolve the tetrazolium salt that the living cells had produced.^40,41^ The absorbance at 570 nm^38^ was measured using a microplate reader. Over the course of a day, various concentrations of gold nanoparticles were evaluated. The culture plate was then loaded with MTT in PBS solution (5 mg ml^−1^) after a specific amount of time. After adding the MTT solution to the wells, the plate was covered with aluminium foil (as MTT is light sensitive) and incubated at 37 °C for at least 3 h.^42^ Following the incubation period, the formation of purple formazan, indicative of cell death, was observed upon examination under a phase-contrast microscope. Subsequently, 100 μl of washing buffer (Hi-media, India) was added to each well, followed by a 4 h incubation at room temperature to dissolve the purple formazan post PBS washing. DMSO was added subsequently. The absorbance at 570 nm and 630 nm was quantified using a BIO-RAD ELISA microplate absorbance spectrophotometer.^43^ Each well was subjected to three absorbance measurements to derive the average, standard deviation, and standard error. Concurrent assays on untreated control sets were also conducted for comparative analysis.

### 2.6 Reactive oxygen species (ROS) detection

HepG2 cells exposed to the individual formulations, such as MQ/GNE,GNP, and the core–shell nano-emulsion MQ/GNE-GNPs were found to produce intercellular ROS using DCF-HDA (2,7-dichloro,dihydro-fluorescin diacetate) dye.^43^ Reactive oxygen species (ROS), such as hydroxyl and peroxyl radicals, were measured in cells using the fluorogenic dye DCF-HDA.^44^ According to how DCF-HDA diffuses within cells, the DCF-HDA assay was performed. Intracellular ROS oxidize it to create the highly fluorescent DCF (2,7-dichloro-dihydro-fluorescein) after cellular esterase has deacetylated it into a non-fluorescent molecule.^45^ Using a fluorescence spectrophotometer, this DCF was investigated at 485 nm for excitation and 535 nm for emission, respectively. After seeding 1 × 10^5^ HepG2 cells per well in a 96-well plate, the cells were cultured in a CO_2_ incubator for a full day. Following that, the cells were exposed to 50 μl ml^−1^ doses of MQ/GNE, GNPs, and MQ/GNE-GNP for 24 h. Following a 24 h treatment period, the cells were rinsed with new medium and incubated with 20 mM of the dye DCF-HDA for 1 h in the dark.^46^ Afterwards, a fluorescence spectrophotometer was used to measure the intensity of DCF fluorescence at 485 and 538 nm for excitation and emission, respectively.^47^

### 2.7 Cell cycle analysis

Flow cytometry is a technique that allows for the quick analysis of individual cells or particles suspended in a buffered salt solution as they pass in front of one or more lasers.^48^ Each particle is examined for one or more fluorescence characteristics and visible light scattering. The first group of techniques uses cell measurements taken at a single time point. This analysis can be multivariate (multiparameter), in which a cell characteristic is assessed in addition to DNA content, or univariate, which is often based on the measurement of the cellular DNA content alone.^17^ The extra characteristic is typically a metabolic or molecular trait that indicates the capacity for cell proliferation or quiescence, or it is connected with the rate at which a cell moves through the cell cycle.^18^ Cells should be grown on a 12-well plate at a density of 1 × 10^5^ cells per millilitre, and they should be incubated with CO_2_ overnight at 37 °C. After the cells have been treated with 50 μl ml^−1^ of MQ/GNE, GNPs, and MQ/GNE-GNP, they were incubated for a full day. To serve as a negative control, one of the wells was left untreated. Following a 24 h incubation period, the medium was collected and stored in a vial, followed by 2 min of trypsinzed cell adherent lysis. The treated cells were subsequently collected and put in the proper vials for storage. The cells were centrifuged for 5 min at 1200 rpm, and the supernatant was disposed of without disturbing the pellet.^49^ The vials were kept cold by being stored in ice. The cells were resuspended in cold PBS and centrifuged at 1200 rpm for 5 min. Next, 3 ml of 70% cold ethanol was added to the sample vials for permeability and fixing. Then, in the vortex mode, ethanol was added to the samples. After that, the samples were incubated with ethanol for 15 min, followed by centrifuging at 1200 rpm for 5 min. Then the ethanol was discarded and 500 μl of PI solution was added.^26^ The samples were again incubated at room temperature for 40 min. Next, 3 ml of PBS was added, and the mixture was centrifuged for 5 min at 1200 rpm. Finally, the supernatant was discarded and a minimum of 1 ml of PBS was added for analysis.

### 2.8 Cell morphology analysis

The cells were cultivated in a 12-well plate at a density of 1 × 10^5^ cells/1 ml and then left overnight at 37 °C in a CO_2_ incubator. The cells were incubated for 24 h after treating them with 50 μl ml^−1^ of MQ/GNE, GNP, and MQ/GNE-GNP in 1 ml of growth medium. To serve as a negative control, one of the wells was left untreated. Afterwards, the cells were harvested straight into centrifuge tubes.^29^ The tubes were then centrifuged for 5 min at 300×*g* and 25 °C, and then the supernatant was carefully discarded, and the cells were washed twice with 1 × PBS. After discarding the PBS entirely, the morphology of the cells was analyzed using a phase-contrast microscope.

### 2.9 Live–dead staining (acridine orange/propidium-iodide assay)

Cell viability was measured using nucleic acid binding dyes, namely acridine orange (AO) and propidium-iodide (PI). As AO is a substance that permeates cells, every nucleated cell that has been stained will display green fluorescence. Since PI only enters cells with broken membranes, dying, dead, and necrotic nucleated cells labelled with the dye will display red fluorescence.^50^ Therefore, when stained with both AO and PI, only live nucleated cells displayed green fluorescence, while the dead nucleated cells displayed red fluorescence. Here, the cells were cultivated in a 12-well plate at a density of 1 × 10^5^ cells/1 ml and then left overnight at 37 °C in a CO_2_ incubator.^51^ After treating the cells with the specific dose of the experimental test chemical in 1 ml of growth media, the cells were incubated for 24 h. To serve as a negative control, one of the wells was left untreated. Afterwards, the cells were harvested straight into centrifuge tubes,^20^ and centrifuged for 5 min at 300×*g* and 25 °C. After carefully decanting the supernatant, 1× PBS was used to wash the cells twice. After discarding the PBS entirely, 500 μl propidium-iodide/acridine orange staining solution was added, ensuring thorough mixing, followed by incubation for 5 min. The cells were then washed with 1× DPBS twice, and then the live/dead cells were counted and immediately imaged using a Carl Zeiss Axio Scope A1 fluorescence microscope (Germany).

### 2.10 Assessment of Wnt/β-catenin expression by flow cytometry

The canonical or β-catenin-dependent branch and the non-canonical or β-catenin-independent branch are the two main branches of the Wnt pathway. The canonical route affects transcriptional control by stabilizing and translocating β-catenin to the nucleus.^52^ On the other hand, the non-canonical route involves many signalling cascades and functions independently of β-catenin. The complex and diverse character of Wnt signalling in the context of liver cancer is influenced by the wide range of Wnt targets and downstream signalling events.^53^ A thorough understanding of this pathway is essential to understand the intricacies associated with hepatocellular carcinoma (HCC). The Wnt pathway plays a crucial role in the initiation of tumours, making it a significant target for therapeutic intervention in HCC.^54^ The molecular mechanisms behind the development and progression of hepatocellular carcinoma are closely linked to mutations and abnormal activation of the Wnt pathway.^55^ Therefore, there is great potential for the creation of novel therapeutic approaches for the treatment of HCC through the focused manipulation of this route. To navigate the intricate web of signalling pathways and eventually block the signalling event that governs tumour growth and survival, combined therapy may be required.

Cells were cultivated in a 12-well plate at a density of 1 × 10^5^ cells/1 ml and then incubated for 24 h at 37 °C in a CO_2_ incubator. After adding the necessary amount of the experimental test substance to 1 ml of growth media, the cells are incubated for a full day. For the purpose of serving as a negative control, one of the wells was left untreated.^56^ Afterwards, the cells were harvested straight into the centrifuge tubes, and then centrifuged for 5 min at 300×*g* and 25 °C.

Next, the supernatant was carefully decanted and washed with 1X PBS twice, and the PBS was then discarded. After 20 min, 0.5 ml of 2% paraformaldehyde solution was added and the cells were further incubated, followed by rinsing with 1X phosphate-buffered saline (PBS) containing 0.5% bovine serum albumin (BSA). In the 0.5% BSA solution, 0.1% Triton-X 100 was added, and the mixture was left to stand for 10 min. Wash twice in 1X phosphate-buffered saline (PBS) containing 0.5% bovine serum albumin (BSA). Add 100 μl of a 1 : 100 dilution of the primary antibody solution, which contains 0.5% of bovine serum albumin (BSA) in 1X phosphate-buffered saline (PBS). Pipette everything well, then let it sit in the dark at room temperature (25 °C) for 60 min. Wash two times in 1X phosphate-buffered saline (PBS) containing 0.5% bovine serum albumin (BSA). Pour in 100 μl of a 1 : 20 dilution of secondary antibody solution, which has 0.5% bovine serum albumin (BSA) in 1X phosphate-buffered saline (PBS).^57^ Pipette everything well, then let it sit in the dark at room temperature (25 °C) for 60 min. Rinse twice with 1X phosphate-buffered saline (PBS) containing 0.5% bovine serum albumin (BSA), thoroughly mix, and then analyse through fluorescent spectroscopy.

### 2.11 Statistical analysis

With the use of Prism 7.0 (Graphpad Software Inc., San Diego, USA), all the data were statistically analyzed. The data are reported as the average ± standard error of at least two different experiments. A comparison test was conducted using one-way ANOVA for the data analysis. A *P* value less than 0.05 indicated that a difference was statistically significant.

## 3. Results and discusion

### 3.1 Formulation of the core–shell nano-emulsion

**Fig. 1:**
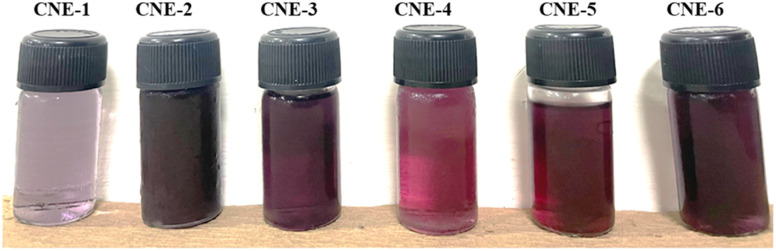
Visual appearance of formulated core–shell nano-emulsions (MQ/GNE-GNP: CNE-1, CNE-2, CNE-3, CNE-4, CNE-5, CNE-6).

The current study used the o/w spontaneous emulsion approach in combination with the nanoprecipitation method to prepare MQ/GNE-coated GNPs for the delivery of mefloquine (MEF). In short, distinct ratios of the core and shell formulations were combined at varying concentrations at room temperature with gentle magnetic swirling until a transparent nano-suspension was formed, with varying co-surfactants investigated for preparation of the nano-emulsion.^26^ To deliver mefloquine to liver cancer cells, we developed a garlic oil nanoemulsion filled with the drug and coated in nanometre-size-based gold nanoparticles. In our previous study, we tried a variety of oils, but garlic was finally selected because of its high drug solubility and capacity to stabilize emulsions. Two surfactants, Cremophore EL and Brij 35 as a cosurfactant, were used to improve the nano-emulsion stability.^58^ Second, as in our previous research, Turkevich's method from 1951 was modified and used to synthesize the GNPs. This method entails reducing a gold salt, usually HAuCl_4_. However, the cationic surfactant CTAB is used as a capping and stabilizing agent in conventional synthesis procedures for producing positively charged gold nanoparticles.^59^ The primary source of gold ions (Au^3+^) is the reduction of chloroauric acid (HAuCl_4_), whereas the bilayer structure containing Au-NPs are produced by cetyltrimethylammonium bromide (CTAB). Because of the adsorbed bilayer, the inner layer, which is bound to the gold surface by its charged head groups, causes a noticeable positive charge on the surface of the nanoparticles (Fig. 1).^18^

Allicin is rich in garlic. One important sulfur molecule found in garlic, allicin, has been demonstrated to have promising anticancer properties.^60^ Many active components, such as arginine, flavonoids, and oligosaccharides, are present in plants high in allium. In contrast to other allium crops, garlic is the focus of most research because to a single compound termed beta-chlorogenic, which facilitates the uptake of glucose by hepatic cells.^61^ First, by attaching to many receptors that are overexpressed on hepatoma cells, the tumour-selective anticancer qualities of garlic can improve a formulation's internalization into cancer cells. Second, a few active metabolites of garlic are likely to be essential for the death of malignant cells due to its multi-targeted effects and lack of significant toxicity. It is possible to improve the solubility, stability, and oral bioavailability of garlic oil by combining it with a nano-emulsion. Third, there is a good chance for surface functionalization with tumour targeting owing to the garlic functional group that is now available.^62^ For the treatment of liver cancer, we have already created size-based gold nanoparticles and an MQ-loaded garlic nano-emulsion (MQ/GNE). While GNP demonstrated an increase in reactive oxygen species and cell permeability, MQ/GNE showed a stronger anticancer impact.

GNPs were created by a reduction process with CTAB and the 99% pure gold chemical salt chloroauric acid (HAuCl_4_·3H_2_O). There are only two possibilities in order to prevent the formulation from becoming more acidic: creating the core–shell nano-emulsion, followed by covering the negatively charged nano-emulsion with the cationic GNP solution *via* electrostatic contact.^63^ The particle size increased when the GNPs were coated with varying nano-emulsion concentrations. These findings were consistent with the core–shell nano-emulsion's size expansion and charge reversal following the coating.

### 3.2 Characterization of the formulated core–shell nano-emulsion (MQ/GNE-GNP)

Different characterization methods were used for the particle morphological and physiochemical characterization, such as DLS, poly dispersity index, zeta potential, TEM (transmission electron microscopy), drug-release kinetics, thermodynamic stability, and FTIR studies (Table 1).

**Table 1:** Particle-size distribution hydrodynamic size (DLS), polydispersity index, and zeta potential of different sizes of core–shell nano-emulsions

CNE(MQ/GNE-GNP)	Hydrodynamic size (nm)	Polydispersity index	Zeta potential (mV)
1	37.8	<0.4	10.4
2	48.5	<0.4	12.2
3	52.1	<0.4	14.3
4	67.9	<0.4	50.5
5	95.9	<0.4	21.8
6	113.8	<0.4	24.9

#### 3.2.1 DLS, polydispersity index, and zeta potential

**Fig. 2:**
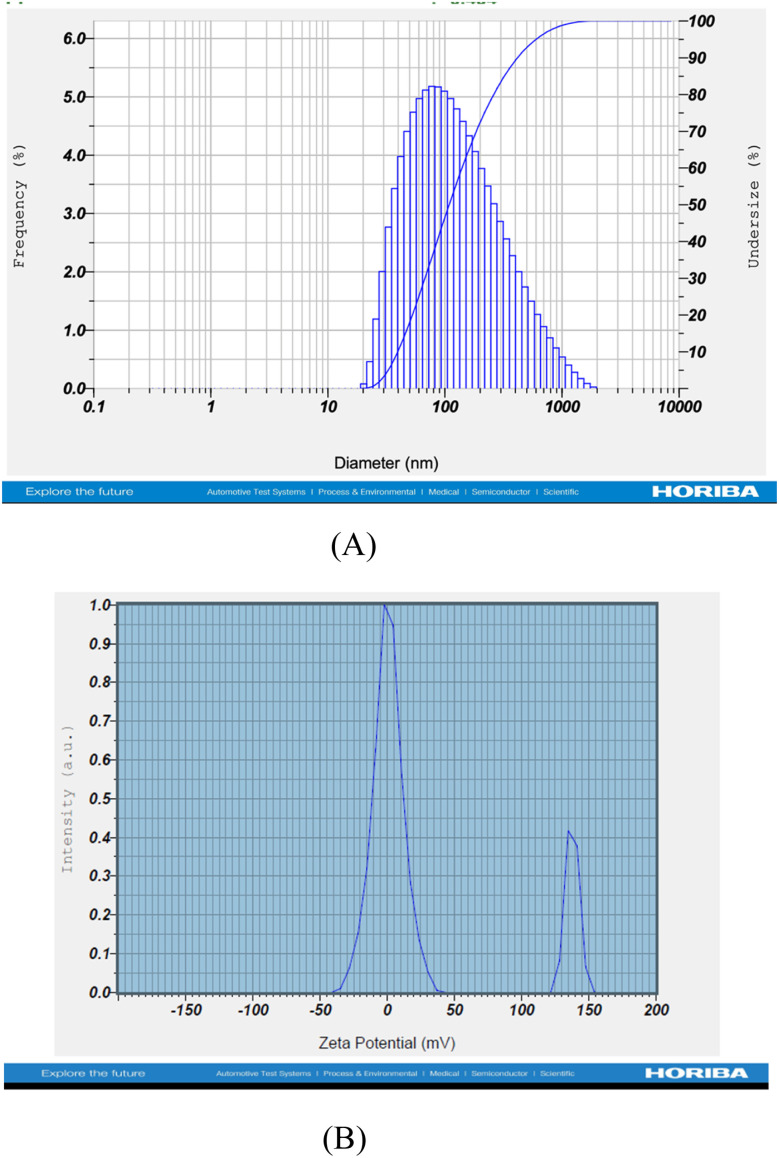
(A) Size-distribution diagram of MQ/GNE-GNP, and (B) zeta potential of MQ/GNE-GNP.

One of the most crucial characteristics of a nanoemulsion for stability testing is droplet size, which also plays an essential role in increasing a drug's bioavailability. One physical characteristic used to comprehend the behaviour of macromolecules based on the charges on a material's surface, and on suspended particles is the zeta potential.^64^ It accelerates the process of predicting long-term stability, and other methods, such as trial formulations, can also be used. From an experimental standpoint, the measured zeta potential's magnitude gives a hint as to a colloidal system's potential stability.^65^ A high zeta potential will signify stability for those particles with very small dimensions, meaning the solution or dispersion will be resistant to aggregation.^65^ The dispersion may fragment and flocculate when repulsive forces are outweighed by attractive forces when the zeta potential is low.^66^ As a result, positive or negative low colloidal particles with a high zeta potential are electrically stable, whereas low zeta potential particles have a propensity to coagulate or flocculate in solution.^67^ Polydispersity ensures consistency and stability of the droplets in a formulation. The homogeneity of the droplet size suffers due to high polydispersity. Its significant findings revealed that AFM and TEM (transmission microscopy) measurements revealed a spherical form of nano-emulsion with a size range of 15 to 70 nm. It discovered that raising the surface charge considerably increases the stability of nano-emulsions due to the repulsive interactions that form between droplets to prevent flocculation and coalescence (Fig. 2).^68^

#### 3.2.2 Thermodynamic stability

Thermodynamic stability results of different oil formulations are shown in Table 2.

**Table 2:** Evaluation of the thermodynamic stability of different oil formulations

Formulation code	Core–shell nano-formulation (MQ/GNE-GNP)		
Different ratios	Centrifugation	Freeze–thawing	Heating–cooling
MQ/GNE-GNP-1	Passed	Fail	Fail
MQ/GNE-GNP-2	Passed	Fail	Fail
MQ/GNE-GNP-3	Passed	Fail	Fail
MQ/GNE-GNP-4	Passed	Passed	Passed
MQ/GNE-GNP-5	Fail	Passed	Passed
MQ/GNE-GNP-6	Fail	Fail	Fail

#### 3.2.3 TEM analysis

**Fig. 3:**
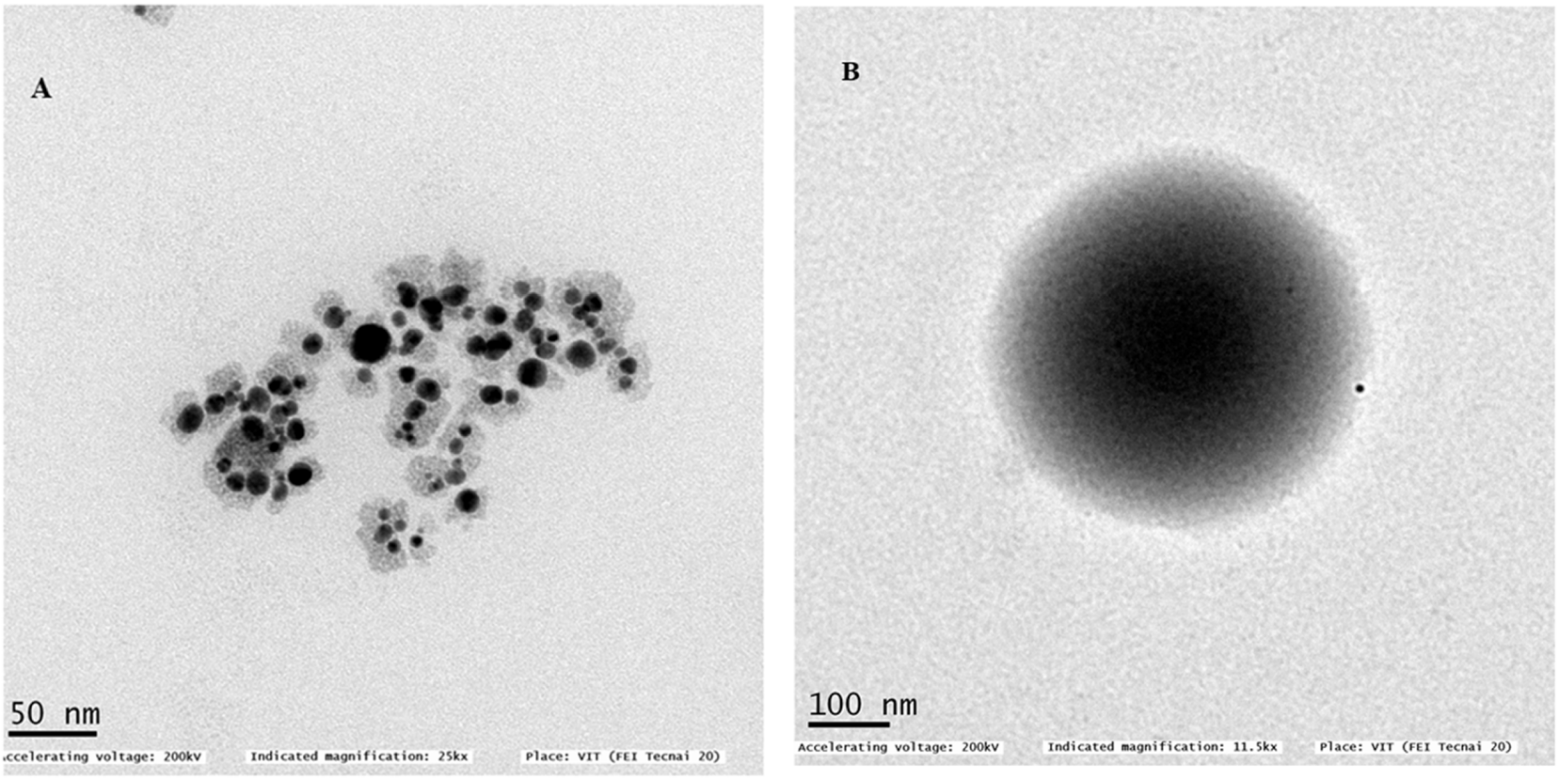
TEM image showing the morphology of MQ/GNE-GNP:MQ/GNE as an oily core and a GNP as the shell at a size of 50 nm (A) and at a size 100 nm (B).

TEM examinations for the prepared core–shell nano-emulsion MQ/GNE-GNP exhibited their spherical shape with the size range of 50–150 nm without any aggregation, which confirmed their excellent colloidal stability, see Fig. 3. Moreover, the TEM images of the MQ/GNE-coated GNP core–shell nano-emulsion showed their core–shell structure, which provides evidence for the efficient coating of the oily core drug-loaded nanoemulsion with a gold nanoparticle as the shell.^26^

#### 3.2.4 FTIR studies

**Fig. 4:**
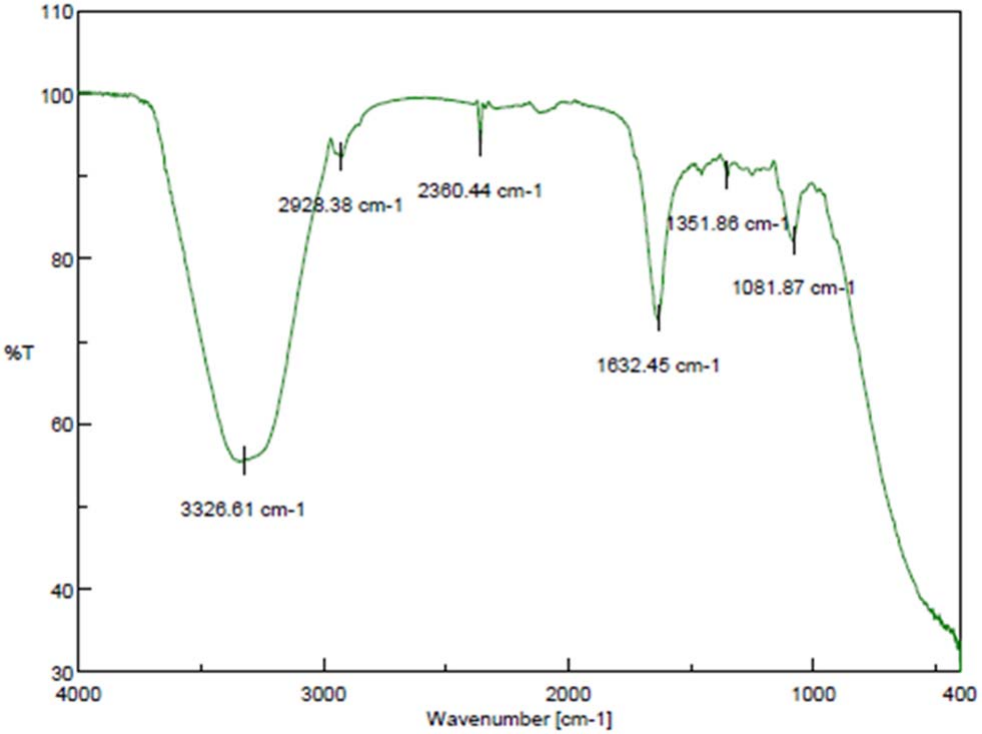
Fourier transform infrared (FTIR) spectra of the core–shell nano-emulsion (MQ/GNE-GNP).

FTIR analysis may support the theory by revealing details about the potential role of functionalized groups in the reduction of MQ/GNE-GNP. There was a significant band representing an end alkyne with C–H stretch at 2928.38–2360.44 cm^−1^, with the C–F bending vibration at 1081 cm^−1^ confirming this^69^ (Fig. 4). In addition, the absorption band of mefloquine was observed at 3326.61 cm^−1^. The characteristic stretching of COO was shown by both symmetric and anti-symmetric peaks at 1632 and 1351 cm^−1^, respectively.^70^ The interaction between GNP and mefloquine/GNE was supported by these results. The nitrogen atoms of mefloquine and the amino acid residues in garlic may form hydrogen bonds within the nano shell, as demonstrated by the N–H stretching vibration peak at 3326.61 cm^−1^.

The stabilizing effect of the nano-emulsion at the surface significantly influences on the formation of spherical MQ/GNE-GNP.^71^ On the other hand, it was previously reported that the GNP functionalized with PEI showed typical carbonyl absorption bands for imide and ester at approximately 3000 and 3400 cm^−1^, respectively.^69^

#### 3.2.5 Stability

**Fig. 5:**
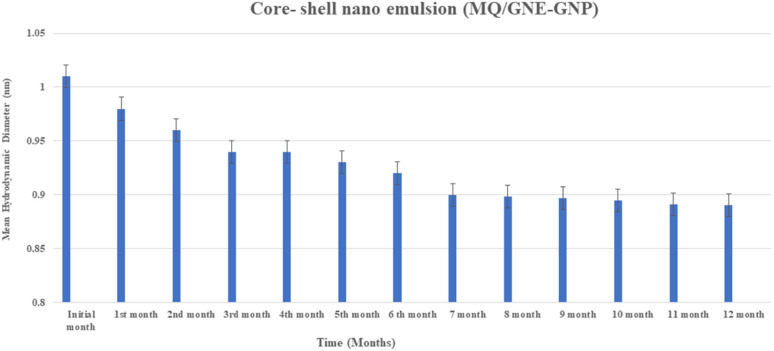
Physical stability of the core–shell nano-emulsion (MQ/GNE-GNP) showing the changes in particle size with time.

The encapsulation of mefloquine within the core–shell nano-emulsion enhanced its stability, shelf life, and oral bioavailability. The prolonged stability of mefloquine in garlic nano-emulsions for up to 12 months underscores the protective efficacy of the core–shell formulation's oil phase for long-term storage.^72^ Efforts were focused on optimizing the solubility, characterization, and stability of garlic-loaded mefloquine nano-emulsions, selected based on their drug-release profile as evaluated with simulated fluids to assess oral bioavailability.^73^ In the graph in Fig. 5, it can be seen that there was no significant changes in particle size. This excellent physical stability of these core–shell formulations can be attributed to using two types of surfactants, namely Cremophore EL and Brij 35, thus providing efficient steric stabilization for the core–shell nano-emulsion.

Furthermore, in addition to the steric stabilization, the cationic GNP shell offers charge-based stabilization. Excellent colloidal stability for dual targeted lactoferrin shell oily core nano capsules has also been reported.

### 3.3 Drug-release kinetics

**Fig. 6:**
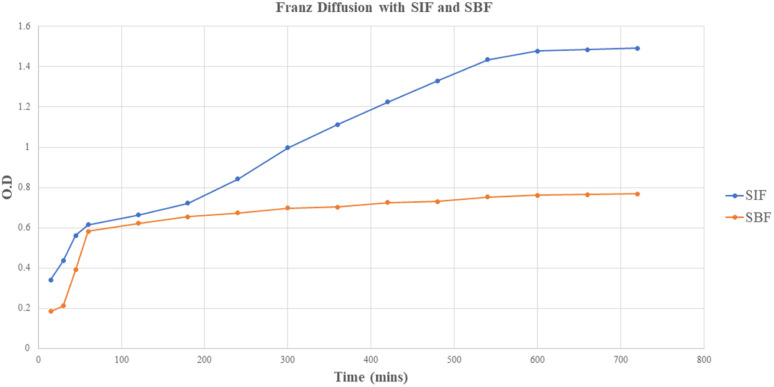
Release profile of the core–shell nano-emulsion (MQ/GNE-GNP) in simulated intestine fluid (SIF) and simulated body fluid (SBF).

The drug-release kinetics of the core–shell nano-emulsion in simulated body fluid (pH 7.4) and simulated intestinal fluid (pH 6.8) are depicted in Fig. 6 using a dialysis membrane. The results showed the release of mefloquine from the core–shell nano-emulsion approximately for 24–48 h. In simulated body fluid, 10–20% release occurred within 3 h, while simulated intestinal fluid showed 40–50% release within 6 h. MQ/GNE-GNP remained in simulated intestinal fluid for up to 24 h, reaching 70–90% saturation, in contrast with 30–35% release in simulated body fluid within 10 h. Mefloquine release in simulated intestinal fluid had an *R*_2_ value of 0.989, indicating high predictability. Notably, 90% release from the garlic oil nano-emulsion occurred within 3 h, with sustained release for up to 24 h, which is vital for maintaining mefloquine levels due to its rapid body metabolism. Garlic oil, containing polyunsaturated fatty acids, offers notable health benefits.^74^ Furthermore, differences in the hydrophobic and hydrophilic properties of the two ligands may also have an impact on the integrity of the outer layer. Owing to the ligands' distinct characteristics, the outer layer would be packed more loosely, enabling the loaded medication to be released more quickly.

This extended period of release was due to the oily core, which served as a drug repository for hydrophobic substances. As a result, GNE-GNP effectively demonstrated a prolonged release of mefloquine, which can raise concerns about safety by preventing blood leakage following intravenous delivery. Additionally, the sequential release pattern can help to increase the anti-tumour activity of mefloquine by sensitizing HCC cells to the drug's release.

### 3.4 *In vitro* evaluation of the therapeutic effects of MQ/GNE, GNPs, and MQ/GNE-GNP against HepG2 cells

We used the MTT assay (short-term cell viability and IC 50 value), reactive oxygen species (ROS) assay, cell morphology cell cycle analysis, cell death analysis by AO/PI staining, and gene expression studies by flow cytometry for *in vitro* evaluation of the therapeutic effects of MQ/GNE, GNPs, and MQ/GNE-GNP against HepG2 cells.

#### 3.4.1 Cytotoxicity studies (MTT assay)

**Fig. 7:**
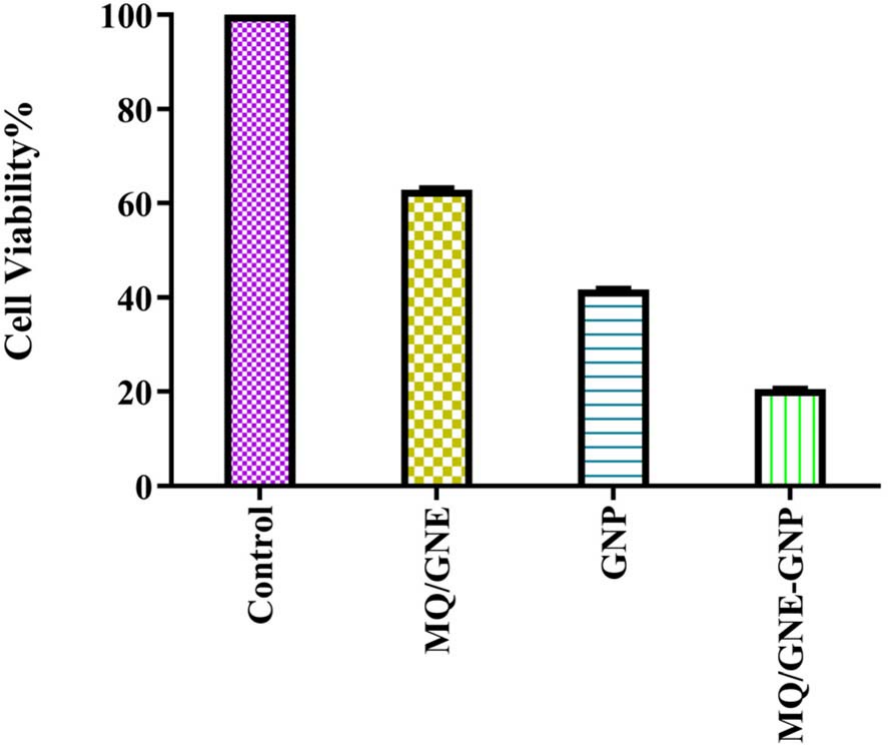
Cytotoxicity analysis of MQ/GNE, GNP and MQ/GNE-GNP on HepG2 at the concentration of 50 μl ml^−1^ after 24 h.

The cytotoxicity of MQ/GNE, GNPs, and MQ/GNE-GNP against HepG2 liver cancer cells was investigated by 3-(4,5-dimethylthiazol-2-yl)-2,5-diphenyl tetrazolium bromide (MTT) assay, see Fig. 7. MQ/GNE-GNP demonstrated high antiproliferative activity towards HepG2 cells with an IC50 value of 50 μl ml^−1^ for 24 h, as also identified in our previous study. According to the viability study, the IC 50 value depended on the dose and interaction time. As stated in the Experimental Section, the confluent or grown cancer cells (HepG2) served as a control, and various concentrations of (50 μl ml^−1^) of MQ/GNE-GNP were exposed for 24 h after being used as treated samples. A predetermined and prescribed MTT assay method was used to examine the cytotoxicity. The gathered information demonstrates that the interaction of MQ/GNE-GNP reduced the viability of HepG2 cells in a dose- or concentration-dependent manner.^17^

According to the MTT assay, the cell viabilities of HepG2 cells treated with MQ/GNE, GNPs, and MQ/GNE-GNP at a concentration of 50 μl ml^−1^ for 24 h were observed to be 65%, 45%, 25% at 24 h (Fig. 7). An important use for core–shell nano-formulations is in combinatorial pharmacotherapy, which combines two or more medications. Core–shell formulations were first applied in anti-tumour combination medication therapy in Sengupta's ground-breaking study.^18^ The efficacy of core–shell lipid nanoparticles containing adriamycin and a P-glycoprotein inhibitor was subsequently shown,^49^ displaying significant inhibitory effects on adriamycin-resistant breast cancer cell lines. We designed a core–shell nano-formulation using GNP in the shell and MQ/GNE in the core for our current study, and we then evaluated the anti-tumour effects of the hybrid system. The proliferation and migration of hepatoma cells were more markedly inhibited by MQ/GNE-GNP, notably.

#### 3.4.2 Reactive oxygen species (ROS assay)

**Fig. 8:**
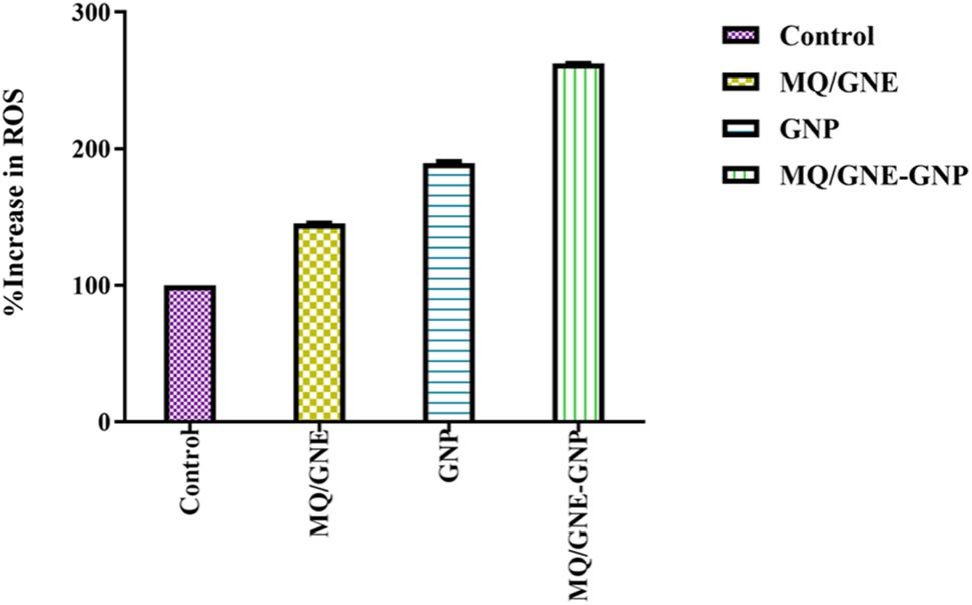
Total ROS generation tests performed on HepG2 cells after exposure to MQ/GNE, GNPs, and MQ/GNE-GNP for 24 h.

In addition, the formation of ROS in HepG2 cells after exposure to MQ/GNE-GNP at a concentration of 50 μl ml^−1^ for 24 hours was examined (Fig. 8). illustrates the rise in ROS levels in cancer cells after exposure to MQ/GNE, GNPs, and MQ/GNE-GNP in comparison to the control. For MQ/GNE, ROS generation in HepG2 cells increased compared to the control at 50 μl ml^−1^. In GNP-treated cells, ROS generation was higher than that in MQ/GNE. Furthermore, its core–shell nano-emulsion (MQ/GNE-GNP) content is high. Different sizes of MQ/GNE, GNPs, and MQ/GNE-GNP can inhibit the proliferation of cancer cells.^75^ It is hypothesized that the various particle sizes and shapes may be able to generate ROS in the cell suspension, which is a crucial component in the formation of free radicals (FRs), which may be able to cross cell membranes and enter the interior of cells.^70^

Additionally, FRs have the capacity to interact with internal organelles, resulting in an enzymatic change and the disorganisation of cells and their contents. The significant role ROS play in the cytotoxicity of nanoparticles (NPs) suggests that excessive generation lowers the cellular antioxidant capacity.^76^ Various physiological and cellular processes, such as inflammation, DNA damage, and apoptosis, are instigated by ROS, including superoxide anion (O^2−^), hydroxyl radical (HO˙), and hydrogen peroxide (H_2_O_2_).^44^ The generation of ROS, as elucidated, stands as a primary mechanistic underpinning the toxicity attributed to nanoparticles (NPs).^43^ Alleviating NP-induced toxicity commonly involves diminishing ROS formation, a goal achievable through the administration of *N*-acetylcysteine (NAC).^45^ Gold nanoparticles may control cancer cell biochemical and enzymatic changes, along with the development and demise of cell organelles.^77^ These results imply the combinational effect whereby different sizes and concentrations can easily enter cells.^78^ In all living things, ROS are constantly being produced. Different metabolic processes that are restricted to the chloroplast, mitochondria, and peroxisomes result in the production of radicals that increase the production of ROS.^79^ When cells are exposed to environmental stresses, like pollutants, pathogens, and light intensity, ROS generation becomes more intense.^80^ Cell damage results from the build-up of oxidizing radicals inside the cell. Due to their smaller size, gold nanoparticles can easily enter cancer cells through diffusion, endocytosis, and/or carrier transport and cause oxidative stress.^81^ It has been reported that reactive oxidative stress plays a key role in the processes that underlie the toxicity of many nanoparticles, either by producing too many reactive oxygen species (ROS) or by reducing the antioxidant capacity of cells. ROS and oxidative stress are implicated in a variety of disorders, most notably cancer, according to growing biochemical, clinical, and epidemiological data.

#### 3.4.3 Cell cycle analysis

**Fig. 9:**
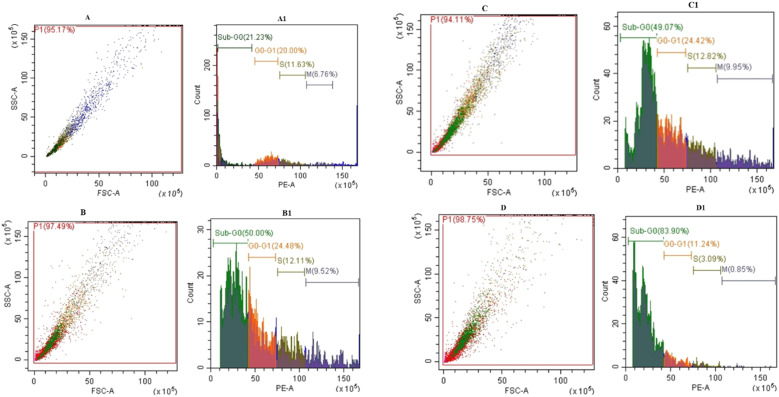
Effects of MQ/GNE, GNP, and MQ/GNE-GNP on the cell cycle distribution in HepG2 cells. (A and A1) represent the control group as untreated, (B and B1) cells treated with MEF/GNE (C and C1) the cells treated with GNP, (D and D1) cells treated with MEF/GNE-GNP.

After a 24 h period, the data showed a statistically significant increase (*p* < 0.05) in the Sub G0 population across all treated cells as compared to the control. Notably, the MQ/GNE-GNP treatment had the largest percentage of the Sub G0 population (83.90%), which was higher than the percentages for MQ/GNE (50.08%) and GNP (49.07%). It is important to note that the cell cycle distribution in G0/G1, S, and M phases did not exhibit any appreciable changes as a result of the treatment with MQ/GNE, GNP, or MQ/GNE-GNP. An increase in the number of cells in the sub G0/G1 phase indicates DNA cleavage and apoptosis. All the treatment groups showed statistically significant (*p* < 0.05) increases in the percentage of cells in the sub G0 peak compared to the control group.^55^ In all the treated groups, this was accompanied by a concurrent decrease in the G0/G1 phase, suggesting that cell development was slowed, resulting in cells entering into the sub G0 phase. Furthermore, since the sub G0 peak is a quantitative marker and unique characteristic of apoptotic cells, the spike in the sub G0 population seen in our study indicates the start of apoptosis.^82^ In addition, the increase in the sub G0 population represents the splitting of nuclear DNA into many pieces, which is suggestive of cell death by triggered apoptosis. DNA fragmentation-induced cell death causes apoptotic cells to have less DNA than healthy cells; this is demonstrated by the appearance of a sub G0 peak in the cell population profile. As described, little damage to DNA usually results in cell cycle arrest, whereas severe and irreversible damage causes a change in the cellular response that leads to cell death *via* apoptosis.^83^ Therefore, the cell cycle profile, which showed an increased sub G0 population, confirmed that Hep G2 cells treated with MQ/GNE, GNP, and MQ/GNE-GNP had undergone apoptosis in this investigation. The aforementioned results align with previously released information. For instance, it has been shown that the sesquiterpene beta-element extracted from *Rhizoma zedoariae* oil inhibits cell viability and induces cell cycle arrest at the sub G0 phase. Comparably, results from a study show that F2S selectively causes cell cycle arrest at the G1 phase of colon cancer and reduces cell survival.^83^ Furthermore, mefloquine was shown to cause cell death and cell cycle arrest in prostate cancer, especially in the sub G0/G1 phase. According to research, A549 cells undergo cell cycle arrest at the sub G0/G1 phase when exposed to gold nanoparticles.^53^ The participation of the Wnt/β-catenin pathway in the cell cycle arrest caused by MQ/GNE-GNP at the sub G0/G1 phase was confirmed by further mechanistic investigations.^52^ In order to obtain a deep comprehension of the exact mechanism of action, thorough mechanistic studies are necessary. Control of the Wnt/β-catenin pathway could be linked to the proven greater efficacy of the core–shell nano-emulsion MQ/GNE-GNP in inducing cell cycle arrest at the sub G0/G1 phase, in comparison to the other two formulations (Fig. 9).

#### 3.4.4 Cell morphology analysis

**Fig. 10:**
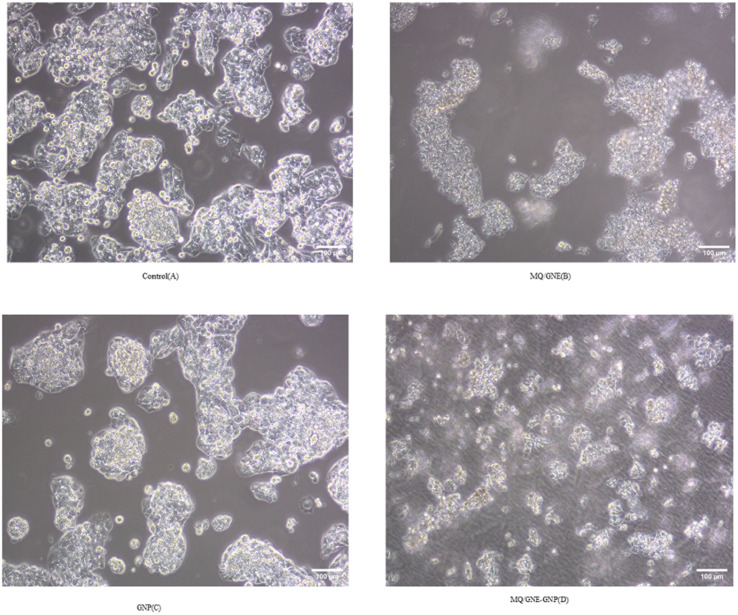
Effects of the core–shell nano-emulsion on the cell morphology in HepG2 cells. (A) Control group (untreated), (B) MQ/GNE, (C) GNP, and (D) MQ/GNE-GNP.

Upon treating cells with various sample materials, including MQ/GNE, GNP, and MQ/GNE-GNP, at comparable concentrations for 24 h, the alterations in cell morphology were examined using a phase-contrast microscope. The impact of these different compounds induced changes in the cell morphology. It can be seen in Fig. 10 that, compared to the control (untreated cells), the treated cells exhibited swelling with blurred edges, disrupted membranes, and reduced density. Additionally, there were fewer contact areas between cells. The impact of MQ/GNE on HepG2 cells was evident, characterized by observable phenomena, such as cellular swelling and rupture. The GNPs influence on HepG2 cells was manifested through membrane disruption, accompanied by a reduction in cellular density.^84^ The combinational core–shell nano-formulation, MQ/GNE-GNP, demonstrated an adverse effect on HepG2 cells, hindering proliferation and accelerating membrane-rupturing cell death. As per the findings of this study, cells thriving under optimal growth conditions and adequate nutrient supply typically exhibited a spindle-shaped morphology, with the distinct filopodia likely facilitating adherent cell proliferation.^85^ In contrast, cells treated with resveratrol assumed a spherical shape, exhibited a loss of crisp filopodia formation, presented a blurred cell membrane boundary, and even displayed a floating phenotype.

#### 3.4.5 Live/dead cell assay(AO/PI staining)

**Fig. 11:**
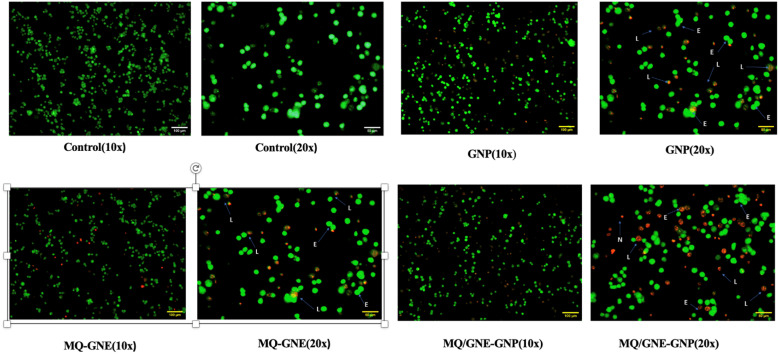
AO/PI staining of untreated (Hep G2 cells) control (10× and 20×), MQ/GNE, GNP, and MQ/GNE-GNP after 24 h. The number of viable cells decreased in MQ/GNE-GNP-treated HepG2 cells and cells went into the apoptosis phase steadily. Morphological changes as observed under a fluorescence microscope AO: Acridine orange, PI: propidium-iodide, E: early apoptotic cells, L: late apoptotic cells.

Depending on the particular bioactive chemical contained in the materials, AO/PI staining of HepG2 cells treated with various compounds showed clearly delineated morphological characteristics suggestive of apoptosis. Significant morphological and colour changes in HepG2 cells treated with MQ/GNE-GNP were evident in the AO/PI-stained pictures, indicating the induction of apoptosis. On the other hand, the untreated cells retained their shapes intact and were green, which is a sign of living cells. There were a few instances of a greenish-red blebbing in the cell membranes of the cells treated with GNPs, MQ/GNE, and the core–shell formulation (MQ/GNE-GNP). Cellular shrinkage, which causes organelles to condense and increases cytoplasmic density, is a characteristic of apoptosis that is especially noticeable in the early stages of the process.^66^ Notably, chromatin condensation is the main feature of early apoptosis, whereas budding, which involves substantial plasma membrane blebbing connected to densely packed organelles, is the main feature of late apoptosis.^17^ All the treated cells showed a considerable rise in the incidence of apoptotic events, indicating a powerful effect of the materials. Simultaneously, early apoptosis and late apoptosis cells were seen in the treated group; these were probably caused by apoptotic cells developing into cell death.^86^ According to the AOPI analysis, at the 24 h point in the therapy, the proportion of necrotic cells showed a noteworthy rise in comparison to the early and late stages of apoptosis. For both multicellular and unicellular eukaryotes, secondary necrosis is the natural end of fully established apoptosis.^20,54^*In vitro*, the lack of phagocytic cells was responsible for the observed development of apoptosis to final disintegration. Furthermore, if failing to clear dying cells quickly, the activation of β-catenin may lead to apoptosis, triggering Wnt signalling and perhaps triggering inflammation and other immunological responses (Fig. 11).

#### 3.4.6 Expression studies of Wnt/β-catenin protein

**Fig. 12:**
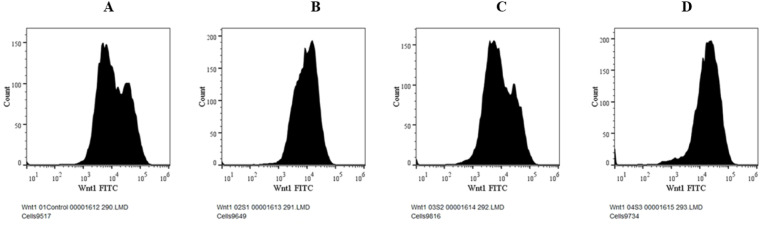
Expression studies of the Wnt1 protein towards HepG2 cells. (A) Control group (untreated), (B) treated with MQ/GNE, (C) treated with GNP, (D) treated with MEF/GNE-GNP for 24 h by flow cytometry (Histogram).

**Fig. 13:**
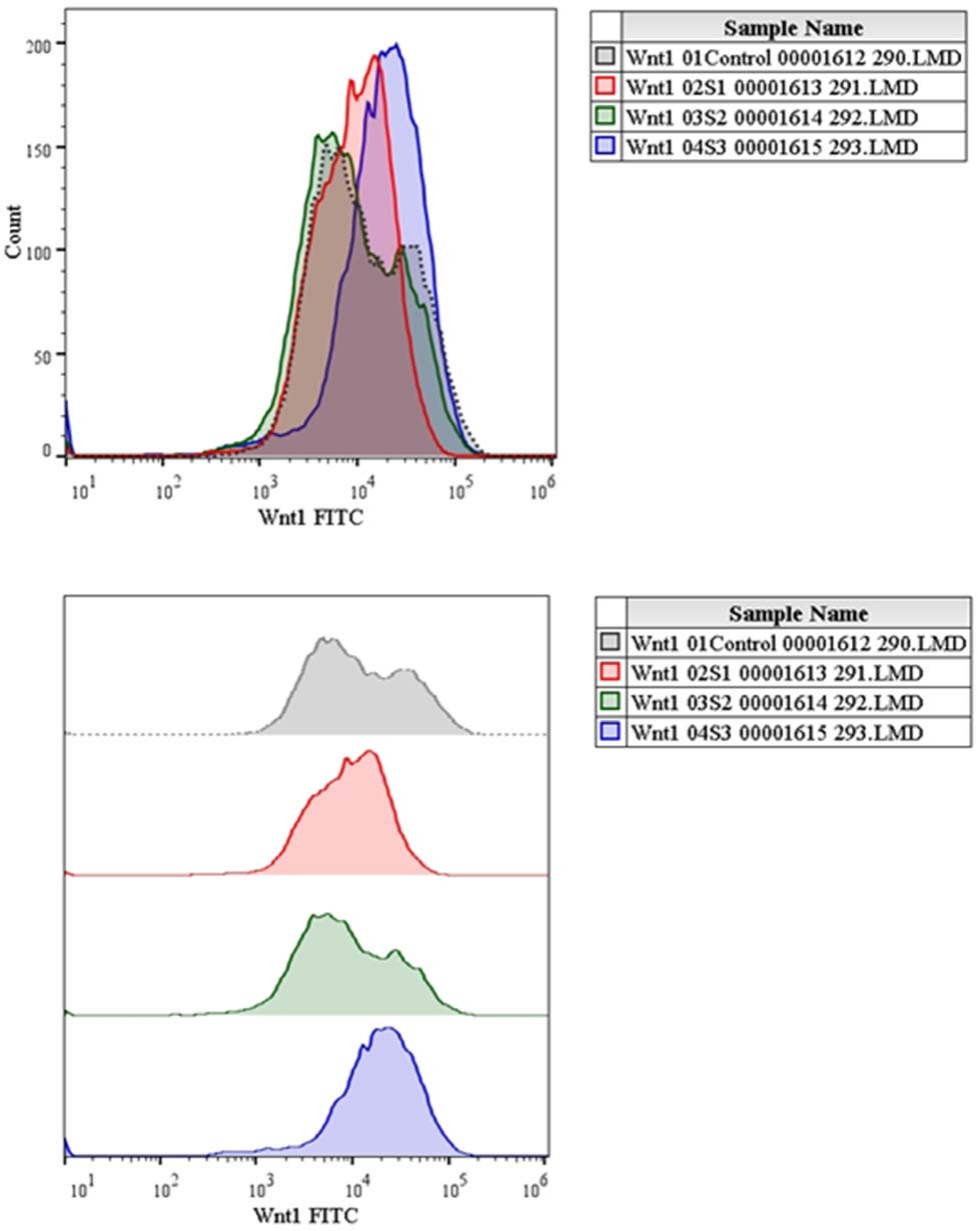
Expression studies of the Wnt1 protein towards HepG2 cells after treatment.

**Fig. 14:**
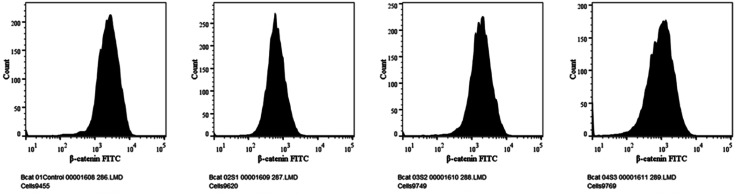
Expression studies of the β-catenin protein towards HepG2 cells.

**Fig. 15:**
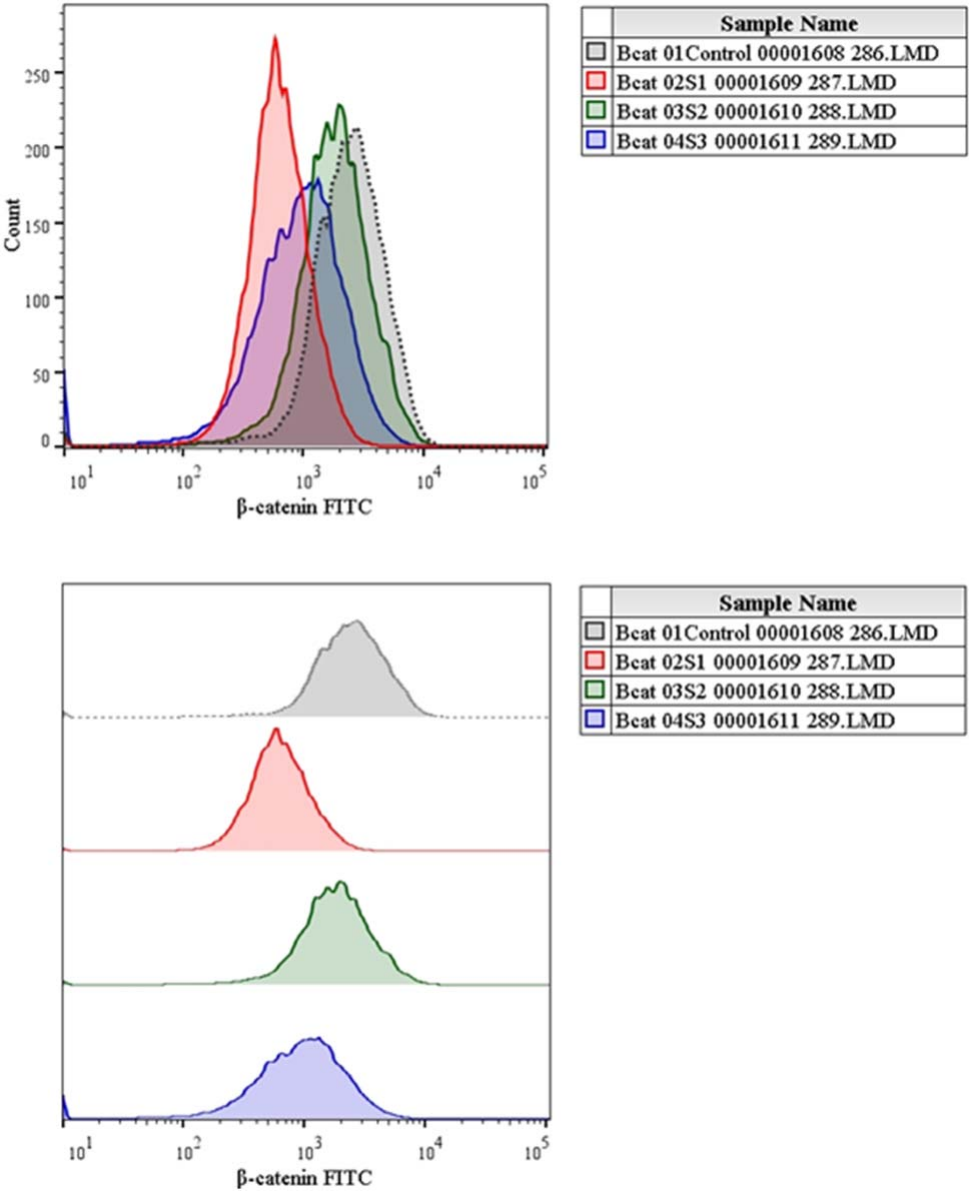
Expression studies of the β-catenin protein towards HepG2 cells.

Numerous cancers, including liver cancer, are known to induce the Wnt/b-catenin signalling pathway.^5^ Additionally, this signalling pathway is essential for controlling proliferation.^1^ In this study, the expressions of Wnt and β-catenin protein were evaluated using flow cytometry analysis. The expression of Wnt with MQ/GNE, GNPs, and MQ/GNE-GNP are shown in the histogram in Fig. 12 while Fig. 13 shows the expression studies of individual samples compared with the control (untreated group). Similarly, the expression of β-catenin with MQ/GNE, GNP, MQ/GNE-GNP are shown in the histogram in Fig. 14 while Fig. 15 shows the expression studies of individual samples compared with the control (untreated group). The histograms show Wnt1 expression in liver cancer cells while treating with different materials, showing that in the overall studies, compared with the control, the expression was less in all the treated groups. In this study on Wnt expression, the cells treated with MQ/GNE and GNPs showed a lower expression compared with the control, while the cells treated with MQ/GNE-GNP showed a higher expression, also compared with the other two compounds, respectively. In terms of the expression of β-catenin with different compounds, the histogram showed that, compared with the protein expression of control cells, the expression of the treated cells protein was higher. However, compared to cells treated with MQ/GNE or GNP alone, cells treated with core–shell nanoemulsion exhibit lower levels of protein expression. A similar pattern is also seen in the fold variations in Wnt and β-catenin expression. In the control group, the fold change is 1, whereas in MQ/GNE-GNP-treated cells, the fold changes are 0.3 and 0.8, indicating reduced protein expression compared to the other two compounds (MQ/GNE and GNP), which show fold changes of 0.8, 0.8, 0.4 and 1.5. These results suggest inhibition of the Wnt/β-catenin pathway, as observed by β-catenin protein inhibition in cells treated with all compounds (MQ/GNE-GNP, MQ/GNE, and GNP). Moderate inhibition of the Wnt pathway is observed with MQ/GNE and GNP treatments. However, the increase in Wnt upon treatment with MQ/GNE-GNP suggested it is able to inhibit β-catenin regardless of Wnt 1 expression, possibly inhibiting the receptors or other proteins involved in the Wnt pathway.

Our findings therefore suggest that the core–shell nano-emulsion MQ/GNE-GNP could be a potential therapeutic target for the prevention of liver cancer. We are interested in further investigating the molecular mechanism by which MQ/GNE-GNP regulates cell death in liver carcinoma. Controlling signalling pathways is another crucial strategy to prevent the growth of tumours.^87^ One of the primary routes for intracellular signal transduction, the Wnt/catenin pathway is involved in several malignant cancers.^88^ Its aberrant activation has a role in the emergence of liver cancer. LRP5/6, Axin, Dvl, and GSK-3 are examples of Wnt/catenin signalling proteins that form transcription cofactors. These cofactors can cause β-catenin to accumulate in the nucleus and stimulate the expression of its target genes, c-myc and survivin, which in turn can cause liver cancer.^89^ Glycogen production is inhibited while intracellular β-catenin expression and kinase activity are increased by the Wnt/β-catenin signalling pathway. In order to control cell cycle and growth, the nucleus can activate the expression of downstream target genes, which catenin can help to enhance.^90^ It indicates that numerous signalling pathways, including Wnt, Hedgehog, Notch, and Bmi, which are traditionally linked to cancer (Fig. 12–15).

We checked the effect of MQ/GNE-GNP on the total, cytoplasmic, and nuclear β-catenin in HepG2 cells and found that the β-catenin levels in these positions were significantly reduced. This indicated the expression and intracellular distribution of β-catenin were regulated by the mefloquine-containing compounds and that MQ/GNE-GNP could inhibit the function of β-catenin, thereby blocking the phosphorylation of β-catenin and preventing its degradation. In order to classify the specific mechanism, this study also detected the related Wnt expression. The result showed that mefloquine-containing MQ/GNE-GNP may reduce β-catenin through downregulation of β-catenin and upregulation of Wnt. Repurposing existing drugs has become a viable approach in the search for novel uses for authorized treatments.^1^ Mefloquine is a medication used for fever that is hydrophobic and has a low solubility in water.^91^ MEF has also recently gained attention as a drug that has been “repositioned” and has shown encouraging anticancer properties. Here, we examined the fundamental mechanisms by which core–shell formulations can reduce HCC *in vitro*. To address the issue of low water solubility, we created a novel core–shell nano-formulation.

## 4. Conclusion

Establishing the existence of a focused therapy poses a special challenge in the treatment of cancer. The efficacy of small compounds and biologics *in vitro* is severely limited by their inadequate distributions, and therapeutic investigations targeting β-catenin are still in their very early stages. Only PRI-724 has progressed to a phase two clinical trial thus far because of its extremely favourable toxicity profile. Yet, there are no reports of employing targeted nanomedicine to downregulate β-catenin in cancer cells precisely. It has recently been proposed that repurposing previously approved clinical medications is a feasible approach for the development of novel cancer nanomedicine. First off, a sizable collection of previously approved FDA drugs still exists that have not been thoroughly investigated for potential application as anticancer agents or as complements to existing cancer treatment modalities. These nanocarriers are designed and fabricated to integrate into the intricate tumour microenvironment, enabling controlled chemical reactions and the spatiotemporal release of drugs. Herein, we demonstrated a compound with mefloquine as a US FDA-approved antimalarial hydrophobic drug loaded with garlic nano-emulsion coated with size-based gold nanoparticle (MQ/GNE-GNP) for the combined delivery of hydrophobic drugs. The combined effect of the core–shell nano-emulsion (MQ/GNE-GNP) demonstrated the sustained release of drugs with excellent physical stability for 12 consecutive months with no significant changes in their particle size. For the developed MQ-loaded garlic nano-emulsion, stability and sustained release were noted in addition to good biosafety. In addition, gold nanoparticles demonstrated enhanced cytotoxicity, elevated reactive oxygen species, and enhanced cell permeability when tested against HepG2 liver cancer cells. When compared to single compounds, like MQ/GNE and GNPs, the superiority of the core–shell nano-emulsion (MQ/GNE-GNP) was demonstrated by the activation of Wnt 1 and the inhibition of β-catenin expression levels. This strategy leverages the effect of the core–shell nano-emulsion, enabling it to achieve a therapeutic window, along with fluorescence imaging and cell cycle analysis as proof-of-concept modalities. Given the versatility of the core–shell nano-emulsion (MQ/GNE-GNP), it can be envisioned that the targeting strategy outlined here can be adopted as an active therapeutic approach against β-catenin in Liver cancer.

## Data availability

Data will be available as per the request.

## Author contributions

Priyadarshini Mohapatra: investigation, methodology, visualization, formal analysis, writing – original draft. N. Chandrasekaran: conceptualization, methodology, supervision, project administration, writing – review and editing.

## Conflicts of interest

The authors involved confirm there are no sources of conflict of interest.
